# Positioning of the Motility Machinery in Halophilic Archaea

**DOI:** 10.1128/mBio.00377-19

**Published:** 2019-05-07

**Authors:** Zhengqun Li, Yoshiaki Kinosita, Marta Rodriguez-Franco, Phillip Nußbaum, Frank Braun, Floriane Delpech, Tessa E. F. Quax, Sonja-Verena Albers

**Affiliations:** a Molecular Biology of Archaea, Faculty of Biology, University of Freiburg, Freiburg, Germany; b Cell Biology, Faculty of Biology, University of Freiburg, Freiburg, Germany; University of Vienna

**Keywords:** archaea, archaellum, cell polarity, chemotaxis, motility

## Abstract

Bacteria and archaea exhibit tactical behavior and can move up and down chemical gradients. This tactical behavior relies on a motility structure, which is guided by a chemosensory system. Environmental signals are sensed by membrane-inserted chemosensory receptors that are organized in large ordered arrays. While the cellular positioning of the chemotaxis machinery and that of the flagellum have been studied in detail in bacteria, we have little knowledge about the localization of such macromolecular assemblies in archaea. Although the archaeal motility structure, the archaellum, is fundamentally different from the flagellum, archaea have received the chemosensory machinery from bacteria and have connected this system with the archaellum. Here, we applied a combination of time-lapse imaging and fluorescence and electron microscopy using the model euryarchaeon Haloferax volcanii and found that archaella were specifically present at the cell poles of actively dividing rod-shaped cells. The chemosensory arrays also had a polar preference, but in addition, several smaller arrays moved freely in the lateral membranes. In the stationary phase, rod-shaped cells became round and chemosensory arrays were disassembled. The positioning of archaella and that of chemosensory arrays are not interdependent and likely require an independent form of positioning machinery. This work showed that, in the rod-shaped haloarchaeal cells, the positioning of the archaellum and of the chemosensory arrays is regulated in time and in space. These insights into the cellular organization of H. volcanii suggest the presence of an active mechanism responsible for the positioning of macromolecular protein complexes in archaea.

## Abstract (precis)

Archaea are ubiquitous single cellular microorganisms that play important ecological roles in nature. The intracellular organization of archaeal cells is among the unresolved mysteries of archaeal biology. With this work, we show that cells of haloarchaea are polarized. The cellular positioning of proteins involved in chemotaxis and motility is spatially and temporally organized in these cells. This suggests the presence of a specific mechanism responsible for the positioning of macromolecular protein complexes in archaea.

## INTRODUCTION

**FIG 1:**
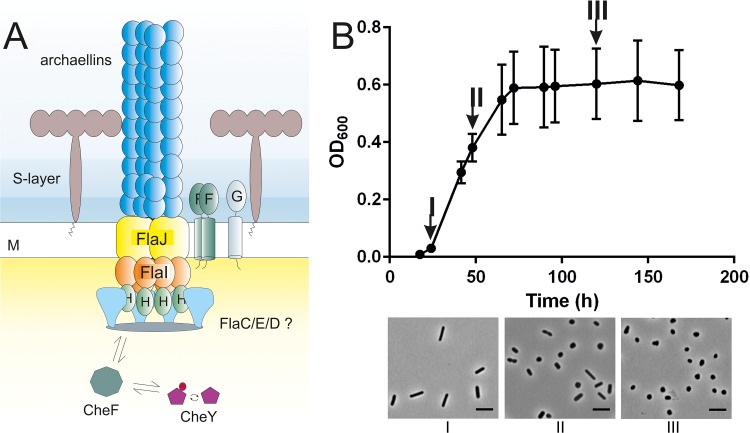
Introduction of the Haloferax volcanii model system. (A) Schematic representation of the archaeal motility structure, the archaellum, based on the cryo-electron microscopy structure described previously (46). The archaeal cell is covered in a surface layer consisting of glycosylated proteins. The archaellum is assembled in a fashion similar to that seen with type IV pili. Assembly and rotation of the filament rely on ATP hydrolysis. Environmental signals are received by chemosensory receptors and lead to phosphorylation of CheY (red circle). CheY-P binds the CheF adaptor protein and travels to the base of the archaellum, where it binds to the archaellum switch complex, which is suggested to consist of FlaC, FlaD, and FlaE (light blue). A switch in the direction of the rotation of the archaellum occurs upon binding of CheY-P. The exact positions of the FlaC, FlaD, and FlaE proteins in the cytosolic ring structure at the lower side of the archaellum motor have not been determined yet. M, cell membrane. (B) (Upper panel) Correlation between the growth phase and cell shape in H. volcanii. The cell shape of the wild-type H. volcanii cells (H26) was analyzed using light microscopy at different time points during a typical growth experiment performed using standard CA medium. (Lower panel) Roman letters below the microscopy images correspond with the time points indicated in the graph in the upper panel. OD_600_, optical density at 600 nm. Scale bar, 2 μm.

Changes in the environment induce a response in microorganisms. The response of motile bacteria and archaea includes directed movement towards more-favorable conditions, a process designated “taxis.” Both bacteria and archaea possess a rotating protein filament to swim through liquid. The two filaments can rotate in either a clockwise or counterclockwise direction (1–3). However, the compositions and structural organizations of these two molecular machines are fundamentally different, and they are designated the “flagellum” in bacteria and the “archaellum” in archaea (Fig. 1A) (4). Interestingly, several archaea possess a chemotaxis system similar to that of bacteria (5, 6). In bacteria, the chemotaxis system is known to direct the rotation of the flagellum by binding to the switch complex at the flagellar motor (7–9). In archaea, the chemotaxis system is responsible for the direction of the rotation of the motility structure and functions in a manner similar to the flagellar system in bacteria (10, 11). Since the structure of the archaellum is fundamentally different from that of the flagellum, the chemotaxis system of archaea requires an adaptor protein to allow communication with the archaellum motor (12, 13).

The chemotaxis system of bacteria and archaea consists of receptors and several different proteins that enable the sensing of temporal gradients. Generally, the receptors, also designated MCPs (methyl-accepting chemotaxis proteins), transfer signals to the histidine kinase CheA (9, 14–16). The interaction between MCPs and CheA is stabilized by the protein CheW (14, 17–19). Autophosphorylated CheA can phosphorylate the response regulator, CheY, which diffuses through the cytoplasm and in bacteria binds the switch complex at the base of the flagellum with higher affinity than unphosphorylated CheY (7–9). In archaea, CheY binds the adaptor protein, CheF, which is hypothesized to mediate the CheY binding to the archaellum motor (Fig. 1A) (12, 13, 20). CheY in both bacteria and archaea is capable of inducing a change in the direction of the rotation of the flagellum and the archaellum, respectively (11, 12, 21, 22). By actively changing the time duration between two reversals, bacteria and archaea are able to bias their movement in a specific direction (23). In addition to the proteins described above, there are several accessory proteins involved in the feedback loops required for the adaptation of the signal (14).

The MCPs are organized in hexagonal arrays together with CheA and CheW (6, 14, 24–29). These large clusters are required for correct signal transduction and signal amplification (27, 30). In bacteria, the arrays, which are composed of transmembrane receptors, are positioned below the (inner) cell membrane, while arrays formed around the soluble receptors are located in the cytoplasm (25). The transmembrane domains of the MCPs of Escherichia coli were previously shown to promote the formation of chemosensory clusters (31).

Arrays of sensory receptors display different positioning patterns across different bacterial species. Often, this positioning pattern is determined by the need to orchestrate the correct inheritance of the functional chemosensory arrays by both daughter cells (32). For example, the well-studied model bacterium E.coli possesses large polar arrays that increase in size with time (33). As a result, the older pole has a larger array than the newer pole. In addition, several smaller clusters are periodically positioned along the lateral membranes and mark the future division sites (34). Recently, it was shown that the lateral clusters of E. coli avoid translocation to the pole regions and, as a result, shuttle continuously between the cell poles during the cell division events that follow (35). In many bacteria, the positioning of the chemosensory arrays at specific cellular sites is an active process that depends on the presence of specific proteins, such as TipN and TipF in Caulobacter crescentus or ParA/MindD homologs in *Vibrio* sp. and Rhodobacter sphaeroides (36–43).

Environmental signals are transferred to the base of the flagellum of the bacteria after processing and amplification at the chemosensory arrays. The positioning of the flagella varies across bacterial species (44). For example, some have multiple randomly positioned flagellae (peritrichous flagellation), while others have multiple flagella at the cell pole (lophotrichous) or only a single flagellum at one (monotrichous) or both (amphitrichous) cell poles (44). The chemosensory arrays and flagella are located at similar sites in some bacteria, and they are positioned at different places in the cells in others. The chemosensory arrays do not have to be placed close to the flagella for efficient signaling, as it was estimated that CheY diffuses over a distance of 1 μm (the length of an average cell) in 1 s (45). Two proteins were identified that control flagellar assembly and placement and the number of flagella in some bacteria; the two proteins were designated FlhF and FlhG, and the latter is a ParA/MinD homolog, such as that described above (44).

Thus, the temporal spatial organization of the chemosensory arrays and flagella is extensively studied in bacteria, and several regulatory mechanisms of these positioning patterns have been identified. In contrast, little is known about the cellular position of the chemosensory arrays and the motility structure in archaea. A few snapshots of the cellular positioning of archaella are available, and they indicated diverse archaellation patterns across the few archaea that have been studied thus far (3, 46–48).

The MCPs of several chemotactic archaea are organized in chemosensory arrays, as is the case for bacteria (6). The chemosensory arrays and the archaellum of Thermococcus kodakaraensis were shown to be connected to a conical frustum, which is localized at the cell poles of the rod-shaped cells (49). However, the spatiotemporal positioning of chemosensory arrays in archaeal cells has not yet been addressed.

We aimed to study the positioning of the important components underlying the tactical behavior in archaea, such as the chemosensory arrays, the response regulator and the archaellum. The recent development of archaeon-adapted fluorescent fusion proteins allows the exploration of archaeal cell biology with live-cell imaging of proteins and macromolecular structures (50). We selected the euryarchaeon Haloferax volcanii as a model because it is the best genetically accessible euryarchaeal system for which fluorescent marker proteins are available (51, 52). In addition, the motile cells are rod-shaped, which facilitates the study of positioning patterns (12, 52). H. volcanii possess a full set of chemotaxis genes, which, like those of all archaeal chemotaxis systems, have more similarity with those from the extensive Bacillus subtilis system than with the streamlined E. coli version (5, 53). Archaea possess the F1 type chemotaxis system, which they likely received from bacteria via horizontal gene transfer (5, 6).

We visualized the localization of the archaellum motor, the chemosensory arrays, and the response regulator using a fluorescence microscopy approach, which indicated active positioning at predefined sites. Live imaging allowed us to follow the archaella and chemosensory arrays during cell division. This work provides the first glimpses of the intracellular organization of the motility machinery in archaea and offers a stepping stone for the further exploration of archaeal cell biology.

## RESULTS

### Positioning pattern of H. volcanii chemosensory arrays.

We studied the positioning of the chemosensory arrays in H. volcanii cells. We selected the adaptor protein CheW as a marker for chemosensory arrays, as this protein has also been used to localize bacterial chemosensory arrays (for example, see Ringgaard et al. [37]). The localization of CheW was studied in H. volcanii cells in the early log phase, as it was previously determined that the cells are motile and rod-shaped under such conditions (12, 52). An example of the correlation between the growth phase and cell shape of H. volcanii cells can be found in Fig. 1B. The cells are rod-shaped in the early log phase, whereas they become pleomorphic/round and nonmotile in the stationary phase (Fig. 1).

**FIG 2:**
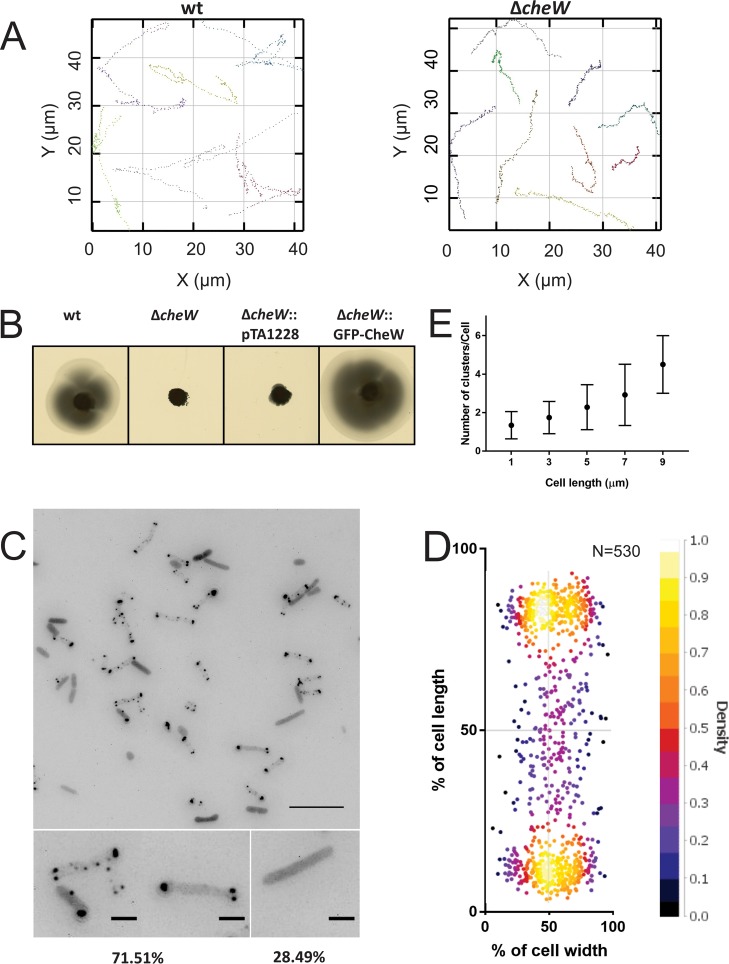
Intracellular distribution of the chemosensory arrays in H. volcanii. (A) A Δ*cheW* strain has altered swimming behavior. Graphs display the *x*-*y* displacement of exemplary swimming cells for each strain. (B) Influence of CheW on directional movement. Results of assays of the motility of different H. volcanii strains on semisolid agar plates are shown. (C) Intracellular distribution of GFP-CheW in H. volcanii Δ*cheW* in the early log phase. The lower panels show closeup views of two different observed distribution patterns, and the numbers at the bottom represent percentages of the total population displaying the distribution (*n* > 1,000). Scale bars, 10 μm (upper panel) and 2 μm (lower panels). (D) Distribution of intracellular clusters. The cluster distances were plotted as a percentage of the total cell length. (E) Dependence of the number of observed intracellular CheW clusters on the cell length. The data were binned at 2-μm intervals. *n* = 530.

Wild-type H. volcanii can form motility rings on semisolid agar plates. The deletion of *cheW* in H. volcanii resulted in the loss of its ability to perform directional movement on semisolid agar plates (Fig. 2B). This result is consistent with a previous study that reported the absence of motility ring formation on a semisolid agar plate in a mutant with a mutation of the H. volcanii *cheW* promoter region (54). Live-cell imaging was applied on a wild-type strain and a *ΔcheW* strain. The H26 wild-type strain was found to display two distinct velocities that likely correspond to forward and reverse swimming (average speeds and standard deviations [SD] of 2.2 ± 0.4 μm s^−1^ and 4.2 ± 1.8 μm s^−1^, respectively) (*n* = 122) (see Fig. S1B in the supplemental material). The analysis of the *ΔcheW* strain showed that the cells were still motile but had an average speed and SD of only 2.5 ± 0.8 μm s^−1^ (*n* = 61). This velocity is significantly different from that seen with wild-type cells (*P = *3.3 × 10^−14^ by Welch’s *t* test (Fig. S1B). The difference might have been due to a loss of the ability to switch the direction of swimming, as the swimming speeds that wild-type haloarchaeal cells display when they swim forward differ from those that they display when they swim backwards (55, 56). The *ΔcheW* cells showed linear movement almost exclusively, with some pauses, while the wild-type cells reversed often (Fig. 2A; see also Fig. S1A in the supplemental material and Movie S1 at https://doi.org/10.6084/m9.figshare.7718456). This demonstrates the importance of CheW for directional movement in H. volcanii.

10.1128/mBio.00377-19.2 FIG S1 Analysis of CheW in H. volcanii. (A) The *ΔcheW* strain showed altered swimming behavior. Graphs display the *x*-*y* displacement of swimming cells for each strain (86 wild-type [wt] cells and 87 *ΔcheW* cells). The *y*-axis data indicate the number of measured switches that fall into each bin. *N*, the number of switching angles. (B) Swimming velocity of the wild-type (*n* = 122) and Δ*cheW* (*n* = 61) H. volcanii strains. The *y*-axis data indicate the number of cells. (C) Run and tumble times of the cells analyzed. Black line, run time; gray line, pause time. *n*, >300. (D) Average diameters of motility rings from different H. volcanii strains measured relative to the wild-type strain in more than three independent experiments, including four biological replicates each. An example of the motility rings is shown in Fig. 1B. (E) The cell length distribution of GFP-CheW expressed by the Δ*cheW* H. volcanii cells used for the analysis whose results are displayed in Fig. 1E. Download FIG S1, TIF file, 1.2 MB. Copyright © 2019 Li et al. 2019 Li et al. This content is distributed under the terms of the Creative Commons Attribution 4.0 International license.

**FIG 3:**
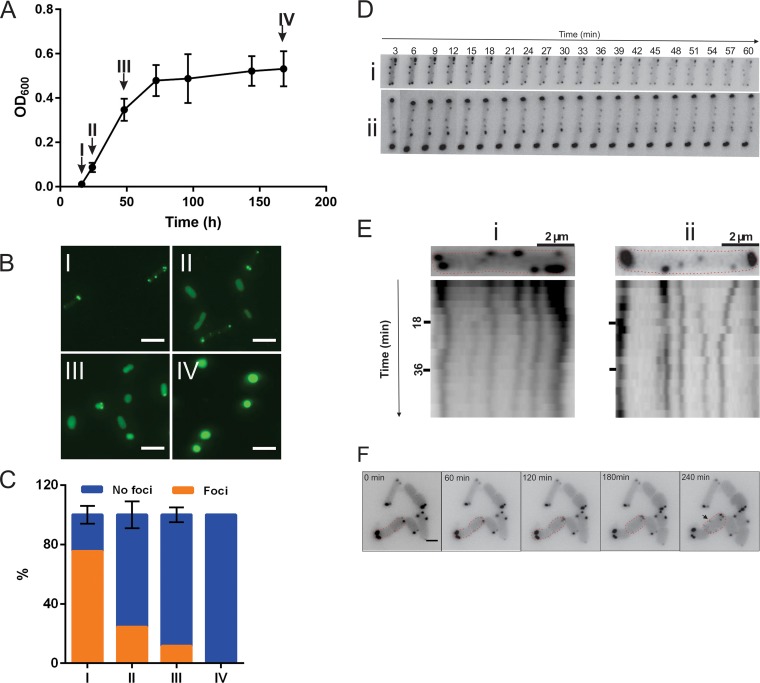
Chemosensory positioning pattern at different growth phases of H. volcanii and mobility of the chemosensory arrays. (A) Growth curve of a typical GFP-CheW-expressing Δ*cheW* H. volcanii strain in CA media. Arrows indicate the time points at which the cells were analyzed as described in the panel B and C legends. (B) Intracellular distribution of GFP-CheW in H. volcanii Δ*cheW* in the growth phases shown in panel A. The Roman numerals correspond to the time points indicated in the graph in panel A. (C) Distribution of the cells described in the panel A legend with intracellular foci versus an equal level of cytoplasmic distribution. Orange, cells with intracellular foci as shown in the left panel in Fig. 2B. Blue, signal evenly distributed in the cytoplasm. *n*, >1,000 per time point. (D) Time-lapse images of two representative cells of a Δ*cheW* strain expressing GFP-CheW showing the mobility of the chemosensory arrays. See also Movie S2 at https://doi.org/10.6084/m9.figshare.7718465. (E) Kymograph of cells displayed in panel C, showing the high mobility of the lateral clusters. (F) Growth and appearance of new chemosensory clusters. Time-lapse images show growing and dividing GFP-CheW-expressing Δ*cheW* H. volcanii cells. See also Movie S3 at https://doi.org/10.6084/m9.figshare.7718471. The arrows indicate newly apparent clusters on the lateral membranes. The red dotted lines indicate cell profiles. Scale bar, 2 μm. Experiments were performed on at least 3 independent occasions.

Next, we expressed N-terminal green fluorescent protein (GFP)-tagged CheW under the control of a tryptophan-inducible promoter in the *ΔcheW* strain and analyzed the results on semisolid agar plates (Fig. 2B; see also Fig. S1D). The wild-type phenotype on semisolid agar plates was able to be restored with the N-terminal GFP fusion of CheW. The fusion protein was correctly expressed, which was confirmed by Western blot analysis using anti-GFP antibodies (Fig. S2). Chemosensory arrays were detected primarily near the cell poles, but smaller clusters were also observed in the lateral membranes (Fig. 2C and D). In ∼30% of the cells, no specific localization could be observed, and the GFP signal was diffuse, suggesting that no large chemosensory arrays were present (Fig. 2C). At one cell pole, one or (often) two large chemosensory clusters were observed (Fig. 2C). Increasing the expression level of GFP-CheW did not significantly change the observed localization pattern, indicating that the observed pattern reflected the natural positioning of the chemosensory arrays (Fig. S3). The number of clusters showed a linear relationship with the cell length, such that longer cells generally harbored greater numbers of chemosensory clusters (Fig. 2E; see also Fig. S1E). This positioning pattern resembles that of the chemosensory clusters in E. coli (34). The localization of the chemosensory arrays was followed during exponential cell growth in liquid culture (Fig. 3A and B). In the early exponential phase, when the cells are rod-shaped and motile, the arrays were present at the cell poles and on the lateral membrane, as shown in Fig. 2C and D. When the cells were entering the late exponential phase or the stationary phase, the number of cells with distinct foci slowly decreased until, near the end, the cells became round and nonmotile, and GFP-CheW showed only diffuse fluorescence in the cytoplasm in all cells (Fig. 3A and B). This indicates that the chemosensory arrays were dismantled in the stationary phase simultaneously with the rounding up of the cells.

10.1128/mBio.00377-19.3 FIG S2 Expression of the GFP fusion proteins as analyzed by SDS-PAGE and Western blot analysis using anti-GFP antibody. Western blot analysis was performed on total cell lysates of exponentially growing cells using the same conditions as those under which fluorescence microscopy was performed. A blot that is representative of at least 3 biological replicates is shown. Download FIG S2, TIF file, 2.0 MB. Copyright © 2019 Li et al. 2019 Li et al. This content is distributed under the terms of the Creative Commons Attribution 4.0 International license.

10.1128/mBio.00377-19.4 FIG S3 The distribution pattern of the GFP fusion proteins analyzed was independent of the expression level. The expression of fusion proteins was induced with different tryptophan (Trp) concentrations in H. volcanii deletion strains of the corresponding genes. The percentage of the cells with intracellular foci was counted and was compared with the percentage of those showing only cytoplasmic localization. The *y*-axis data represent the percentages of the cells versus the total. *n*, >500. Download FIG S3, TIF file, 0.8 MB. Copyright © 2019 Li et al. 2019 Li et al. This content is distributed under the terms of the Creative Commons Attribution 4.0 International license.

To analyze the mobility of the chemosensory clusters, time-lapse imaging was applied (see Movie S2 at https://doi.org/10.6084/m9.figshare.7718471). Both polar and lateral clusters were dynamic, but the movement of the polar clusters seemed restricted to the polar region (Fig. 3C and D). In addition, we often observed fission and fusions of both the polar and lateral clusters (see Movie S2 at https://doi.org/10.6084/m9.figshare.7718471). The polar clusters in E. coli are also dynamic; however, in contrast to H. volcanii, lateral chemosensory clusters are nearly immobile in this bacterium (34).

Next, the appearance of new clusters was analyzed during cell growth and division (Fig. 3E; see also Movie S3 at https://doi.org/10.6084/m9.figshare.7718471). Rod-shaped H. volcanii cells divide at the middle of the cell. New chemosensory clusters were formed regularly and appeared predominantly at random positions along the lateral membranes (Fig. 3E). However, after several rounds of cell division, these lateral clusters eventually became polar. In addition, new clusters were also occasionally observed in the polar region.

### Archaella are localized at the cell poles of H. volcanii.

**FIG 4:**
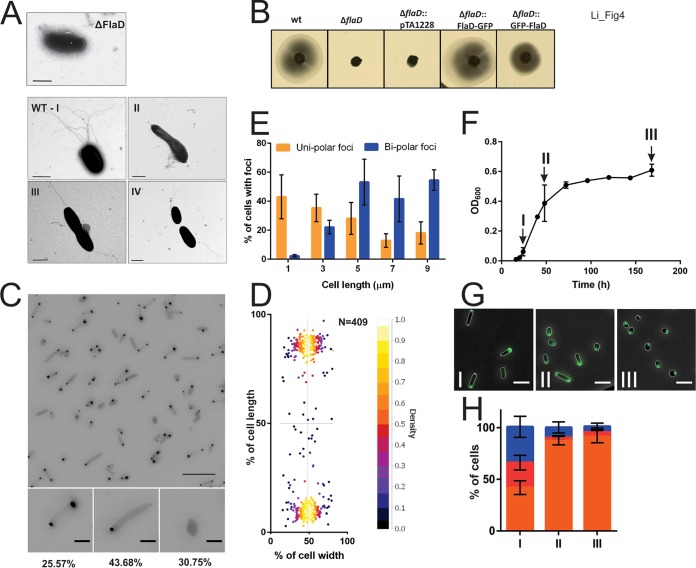
Cellular distribution of the archaella in H. volcanii. (A) Transmission electron microscopy of the wild-type and Δ*flaD* H. volcanii cells in exponential growth phase. The four Roman numerals indicate the different archaellum distribution patterns that were observed for the wild-type (WT) cells as follows: I, archaella at one cell pole; II, elongated cell with archaella at both cell poles; II, constricting cell with archaella at both cell poles; IV, two cells, each with archaella at one pole. WT, wild type. Scale bar, 1 μm (B) Influence of FlaD on directional movement. The panels show results of assays of the motility of different H. volcanii strains on semisolid agar plates. (C) Intracellular distribution of FlaD-GFP in H. volcanii Δ*flaD* in the early log phase. The lower panels show closeup views of two different observed distribution patterns, and the numbers at the bottom represent percentages of the total population displaying the distribution (*n*, >1,000). Scale bars, 10 μm (upper panel) and 2 μm (lower panels). (D) Distribution of intracellular clusters. The cluster distances were plotted as a percentage of the total cell length. (E) Dependence of the number of observed intracellular FlaD foci on the cell length. The data were binned at 2-μm intervals. Error bars, SD. (F) Growth curve of a typical FlaD-GFP-expressing Δ*flaD* H. volcanii strain in CA media. Arrows indicate the time points at which the cells were analyzed as described in the panel G and H legends. (G) Intracellular distribution of GFP-CheW in H. volcanii Δ*cheW* in the growth phases as described in the panel F legend. The Roman numerals correspond to the time points indicated in the graph in panel F. (H) Distribution of the cells described in the panel G legend with the intracellular foci versus an equal level of cytoplasmic distribution. Orange, unipolar foci; red, bipolar foci; blue, signal evenly distributed in the cytoplasm. Error bars, SD. *n*, >1,000 per time point. Experiments were performed on at least 3 independent occasions.

The archaellum is responsible for the swimming movement of archaea in liquid media. To map the cellular positioning of the archaellum, we first imaged wild-type H. volcanii cells using transmission electron microscopy (TEM) in the early log phase. During this growth phase, the cells are typically rod-shaped and mostly have archaella (Fig. 4A). We observed unipolarly or bipolarly archaellated cells. Elongated cells and cells that were starting to constrict at mid-cell were generally bipolar and archaellated (Fig. 4A). The different archaellation patterns observed are shown in Fig. 4A.

Euryarchaea encode the archaellum proteins FlaC, FlaD, and FlaE (see model Fig. 1A). These proteins are absent from the archaellum operons of crenarchaea, which instead encode FlaX, that forms a ring structure at the base of the archaellum motor (4). It has been suggested that the euryarchaeon-specific FlaC, FlaD, and FlaE proteins might form a complex at the base of the archaellum of euryarchaea, where they would be able receive signals from the chemotaxis system, similarly to the bacterial switch complex (Fig. 1A) (13, 46, 49). Since these proteins are suggested to be located at the periphery of the archaellum motor, we hypothesized that they might be excellent candidates for fusion with the fluorescent proteins without disturbing the functioning of the archaellum motor. Therefore, we studied the subcellular positioning of the archaella using FlaD as a marker (Fig. 4). We first deleted *flaD* in H. volcanii and observed that the cells were nonmotile on semisolid agar plates (Fig. 4B), which is consistent with the preliminary results from a *ΔflaD* strain in Halobacterium salinarum (57). As expected, analyzed using time-lapse microscopy, the cells appeared to be nonmotile in liquid media (data not shown). An analysis performed with TEM indicated that the archaella were generally not present at the surface of a Δ*flaD* strain (Fig. 4A).

10.1128/mBio.00377-19.5 FIG S4 FlaD distribution pattern. (A) Average diameters of the motility rings from different H. volcanii strains measured relative to the wild type (WT) in more than three independent experiments, including four biological replicates each. An example of the motility rings is shown in Fig. 3A. (B) Cell length distribution of the FlaD-GFP-expressing Δ*flaD* H. volcanii cells used for the analysis whose results are displayed in Fig. 3E. Download FIG S4, TIF file, 0.7 MB. Copyright © 2019 Li et al. 2019 Li et al. This content is distributed under the terms of the Creative Commons Attribution 4.0 International license.

Expression of the FlaD-GFP fusion protein completely restored the defective motility of a Δ*flaD* strain (Fig. S4). The fusion protein was correctly expressed as observed by Western blot analysis (Fig. S2). FlaD-GFP was observed primarily at the cell poles (Fig. 4C and D). Approximately 44% of the population consisted of unipolarly archaellated cells, and 26% were bipolarly archaellated cells (Fig. 4C). No cells with more than two foci or with foci on the lateral membrane were observed, which suggests that the archaella are usually present exclusively at the cell poles. Longer cells generally contained archaella at both poles, while smaller cells were archaellated at one pole (Fig. 4E and S4). These findings are consistent with our observation of archaellated wild-type cells by the use of transmission electron microscopy (TEM) (Fig. 4A) and suggest that when the cells elongate just before cell division, they are bipolarly archaellated. Usually, the signal was more intense at one cell pole than at the other pole (Fig. 4C). The positioning pattern of FlaD was not significantly affected by different levels of expression of the protein (Fig. S3).

During the early log phase of an exponentially growing liquid culture, the cells displayed a unipolar or bipolar FlaD signal, as shown in Fig. 4. Upon entering the late exponential and stationary phases, the number of cells with two FlaD foci slowly decreased until, near the end, the GFP-FlaD signal was observed only as a single focus at the membrane of the round cells (Fig. 4F and G and H). This might indicate that the cells that were actively dividing in the early log phase resulted in a relatively high number of bipolarly archaellated cells. Later, in the stationary phase, the cells usually contain the archaellum motor at only one cell pole or at one position with a round cell.

**FIG 5:**
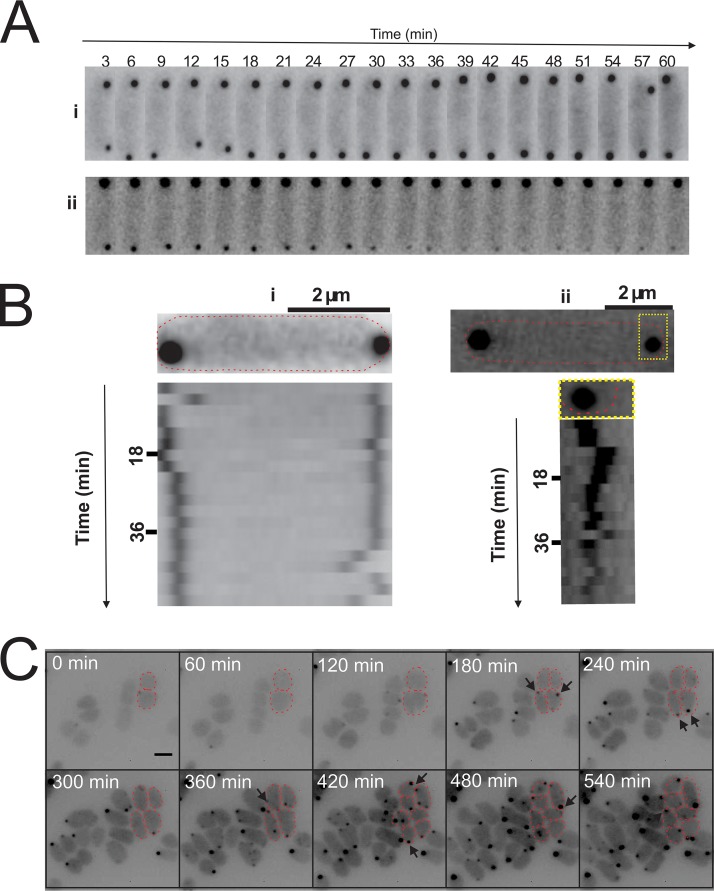
Mobility and new appearance of the FlaD clusters. (A) Time-lapse images of two representative cells of a FlaD-GFP-expressing Δ*flaD* strain showing the mobility of FlaD clusters. See also Movie S4 at https://doi.org/10.6084/m9.figshare.7718480. (B) Kymograph of the cells displayed in panel A showing the high mobility of the FlaD clusters. (C) Growth and appearance of new chemosensory clusters. Time-lapse images of growing and dividing FlaD-GFP-expressing Δ*flaD* H. volcanii cells are shown. The arrows indicate newly apparent clusters. See also Movie S5 at https://doi.org/10.6084/m9.figshare.7718480.

Time-lapse analysis of the cells expressing FlaD-GFP showed that the foci formed by the FlaD proteins were dynamic and moved freely in the polar region (Fig. 5A). In particular, the smaller foci showed a substantial amount of movement, while the larger FlaD foci remained mostly at the same position in the cell polar region. Occasionally, movement of the small FlaD foci from one cell pole to another was observed, which indicates that the small foci could have represented a prearchaellum motor complex that was not yet firmly anchored in the membrane (see Movie S4 at https://doi.org/10.6084/m9.figshare.7718480). The movement was discontinuous, and there were phases of high mobility, followed by phases of lower mobility. The observation of the dividing cells revealed that the formation of new FlaD foci during growth and cell division occurred around the cell poles; however, as the foci remained dynamic, the newly appearing foci were not always strictly polar (Fig. 5C; see also Movie S5 at https://doi.org/10.6084/m9.figshare.7718495). Eventually, the foci clearly became polar during the next rounds of cell division (Fig. 5C; see also Movie S5 at https://doi.org/10.6084/m9.figshare.7718495). Thus, the archaella are present at the poles of actively dividing H. volcanii cells. The very dynamic small FlaD foci could have represented FlaD precomplexes that, once docked to the archaellum motor, stayed more stably positioned at the cell pole.

### The archaeal response regulator CheY shuttles between the chemosensory arrays and the archaellum motor.

After determining the subcellular positioning of the chemosensory arrays and the archaellum, we then focused on the proteins responsible for communication between these macromolecular complexes. In archaea, CheY-P requires the adaptor protein CheF to bind to the archaellum motor (10–13).

**FIG 6:**
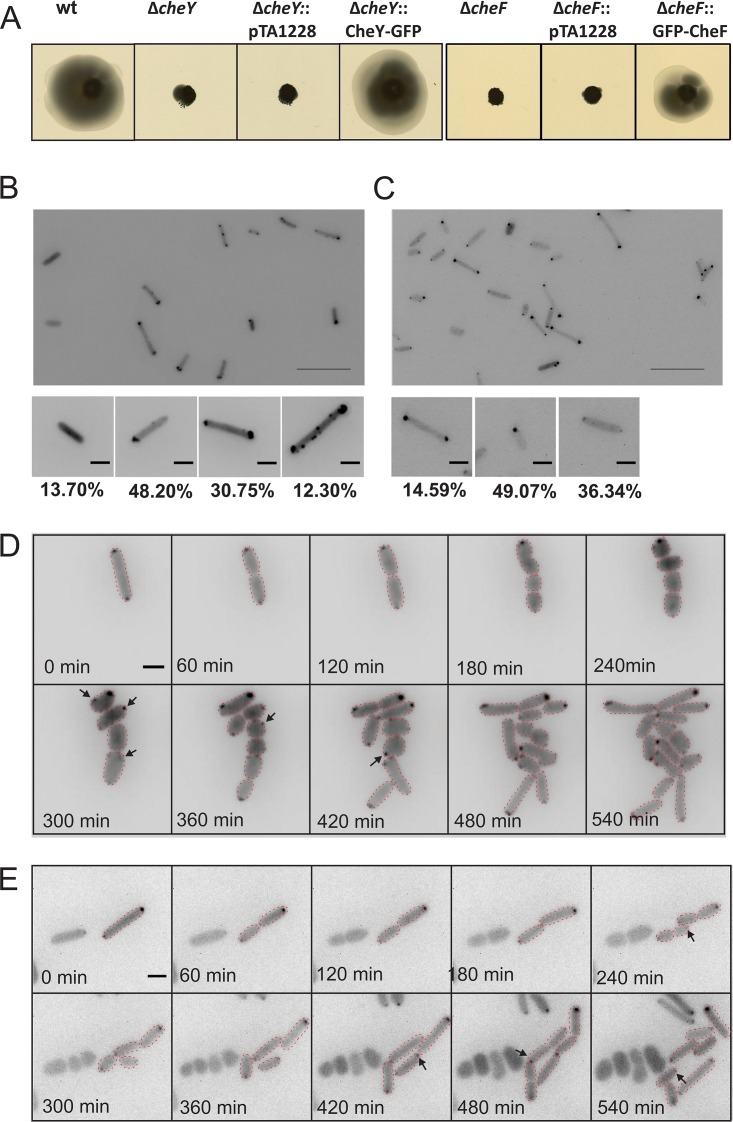
Intracellular distribution and mobility of the CheY response regulator and the CheF adaptor protein in H. volcanii. (A) Influence of CheY and CheF on directional movement. The panels show results of assays of the motility of different H. volcanii strains on semisolid agar plates. pTA1228, empty plasmid. (B and C) Intracellular distribution of CheY-GFP in H. volcanii Δ*cheY* (B) and of GFP-CheF in H. volcanii Δ*cheF* (C) in the early log phase. The lower panels show closeup views of two different observed distribution patterns, and the numbers at the bottom represent percentages of the total population displaying the distribution (*n*, >1,000). Scale bars, 10 μm (upper panel) and 2 μm (lower panels). (D and E) Time-lapse images of dividing CheY-GFP-expressing Δ*cheY* H. volcanii cells (D) and of GFP-CheF expression in Δ*cheF* H. volcanii cells (E). Arrows indicate newly apparent foci. Scale bar, 2 μm. See also Movies S6 and S7 at https://doi.org/10.6084/m9.figshare.7718486 and https://doi.org/10.6084/m9.figshare.7718495.

A Δ*cheY* H. volcanii strain is not capable of directional movement on semisolid agar plates because it can no longer change the direction of the archaellum rotation (12). We expressed GFP fusion proteins in a Δ*cheY* background and found that CheY-GFP could restore the directional movement on semisolid agar plates (Fig. 6A; see also Fig. S5A). Western blot analysis showed that the full-length fusion proteins were expressed correctly (Fig. S2). CheY-GFP was detected in the cytoplasm, but the signal was more intense around the cell poles (Fig. 6B; see also Fig. S5C). The CheY-GFP signal was unipolar (48% of cells) or bipolar (31% of cells). Sometimes (in ∼12% of cells), smaller foci along the lateral membranes were also detected, similarly to what was observed during the GFP-CheW expression (Fig. 6B). The positioning patterns of CheY remained similar at different levels of expression of the protein (Fig. S3). The CheY foci showed some mobility, and the lateral clusters in particular were dynamic, similarly to the results seen with the chemosensory arrays (see Movie S6 at https://doi.org/10.6084/m9.figshare.7718486). The observation of the dividing cells showed that the new CheY foci emerged at positions close to the cell poles (Fig. 6D; see also Movie S7 at https://doi.org/10.6084/m9.figshare.7718495). The positioning pattern of CheY shares characteristics with the patterns of both CheW and FlaD, as could be expected from a protein that transfers signals between the chemosensory arrays and the archaellum motor complex.

10.1128/mBio.00377-19.6 FIG S5 Expression and distribution of the GFP fusions of the CheY response regulator and the CheF adaptor protein. (A and B) Quantification of the motility halos on the semisolid agar plates shown in Fig. 6A. Data represent the average diameters of the motility rings from different H. volcanii strains measured relative to the WT in more than three independent experiments, including four biological replicates each. (C and D) Distribution of intracellular CheY-GFP clusters (C) and GFP-CheF clusters (D) from the experiments whose results are shown in Fig. 6B and C, respectively. The cluster distances were plotted as percentages of the total cell length. Download FIG S5, TIF file, 1.2 MB. Copyright © 2019 Li et al. 2019 Li et al. This content is distributed under the terms of the Creative Commons Attribution 4.0 International license.

As described above, CheY requires the adaptor protein CheF to connect to the archaellum. In a fashion similar to that used for analysis of CheY, we studied the cellular positioning of CheF. H. volcanii possesses two *cheF* genes, and it was previously shown that *cheF*, in contrast to *cheF2*, has a major effect on the directional movement (12, 13); thus, we focused on the cellular positioning of *cheF*. The expression of GFP-CheF in a Δ*cheF* strain was able to restore the wild-type phenotype, as observed on semisolid agar plates (Fig. 6A; see also Fig. S5B). Western blot analysis showed that the full-length fusion proteins were correctly expressed (Fig. S2). The expression of the fusion protein led to diffuse fluorescence in the cytoplasm and to the presence of a higher signal around the cell poles, as was the case for CheY. CheF was localized at unipolar sites (14% of cells) or at bipolar sites (49% of cells), or it was diffuse in the cytoplasm (36% of cells) (Fig. 6C). Smaller foci along the lateral membrane, as in the case of CheY, could not be clearly distinguished, as the fluorescence signal was weak. The CheF positioning patterns remained similar at different levels of expression of the protein (Fig. S3). Time-lapse imaging showed that the polar clusters displayed only limited mobility (see Movie S8 at https://doi.org/10.6084/m9.figshare.7718495). Newly formed foci were first found in the vicinity of the cell poles (Fig. 6E; see also Movie S9 at https://doi.org/10.6084/m9.figshare.7718507.v1). Thus, the CheF positioning patterns, similarly to those of CheY, shared characteristics with both those of the archaella and those of the chemosensory arrays.

### Positioning of the chemosensory arrays and positioning of the archaellum are not linked.

**FIG 7:**
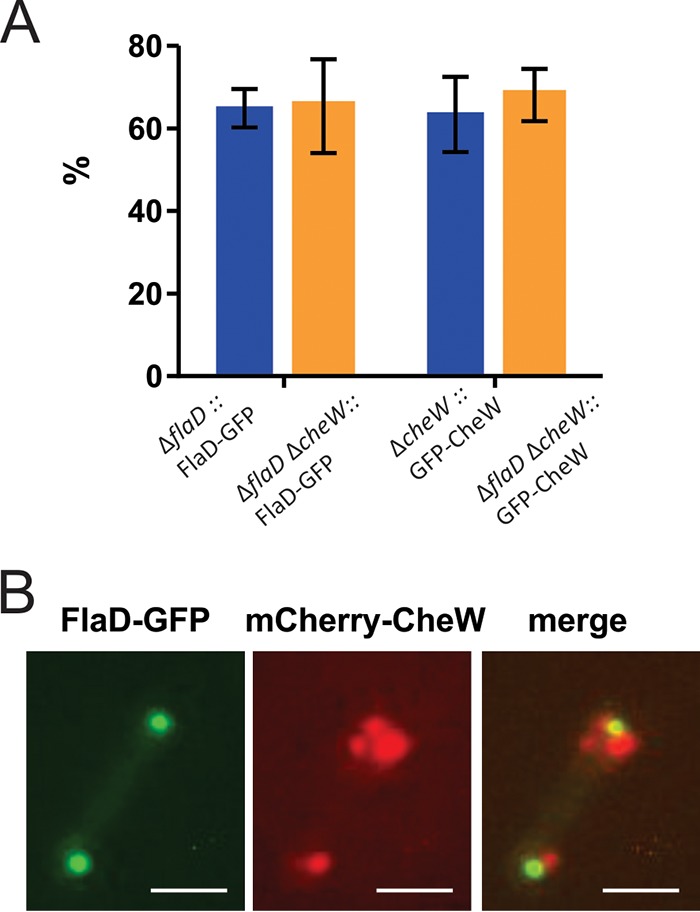
The positioning of the chemosensory arrays and the positioning of the archaella are not interdependent. (A) Percentages of cells with intracellular foci in single-knockout and double-knockout strains. The positioning patterns of the single and double knockouts were not significantly different, indicating that FlaD and CheW can localize independently of each other. (B) Colocalization of FlaD-GFP and mCherry-CheW in the Δ*flaD* Δ*cheW* background. Scale bar, 2 μm.

Since we observed that the chemosensory arrays and archaella were both found primarily at the cell poles of H. volcanii, we aimed to assess if the positioning of each was dependent on that of the other. We created a Δ*flaD* Δ*cheW* strain and expressed GFP-CheW in the Δ*flaD* Δ*cheW* strain, which showed that the chemosensory arrays were still localized at the cell poles and in the absence of FlaD (Fig. 7A; see also Fig. S6A). Similarly, the expression of FlaD-GFP in the Δ*flaD* Δ*cheW* background showed that FlaD was still positioned at the cell poles, with a positioning pattern similar to that seen with the Δ*flaD* strain (Fig. 7A; see also Fig. S6B). When mCherry-CheW and FlaD-GFP were expressed in the double-knockout strain, FlaD and CheW were still both primarily localized at the cell poles, as was the case in the single knockouts (Fig. 7B). However, the regions of FlaD and CheW localization did not overlap or overlapped only partly (Fig. 7B). Occasionally, the positioning of the archaella and chemosensory arrays was also observed at different cell poles. Thus, the chemosensory arrays and archaella are both positioned at the cell poles of the rod-shaped species H. volcanii, but their positions are not interdependent and instead are likely to depend on one or two independent positioning mechanisms.

10.1128/mBio.00377-19.7 FIG S6 The positioning of the archaella and the positioning of the chemosensory arrays are not interdependent. (A) Expression of GFP-CheW in a Δ*cheW* Δ*flaD* H. volcanii strain in the early log phase. (B) Expression of FlaD-GFP in a Δ*cheW* Δ*flaD* H. volcanii strain in the early log phase. The lower panels show closeup views of two different observed distribution patterns, and the numbers at the bottom represent the percentages of the total population displaying the distribution (*n* > 1,000). Scale bars, 10 μm (upper panel) and 2 μm (lower panels). Download FIG S6, TIF file, 2.3 MB. Copyright © 2019 Li et al. 2019 Li et al. This content is distributed under the terms of the Creative Commons Attribution 4.0 International license.

## DISCUSSION

**FIG 8:**
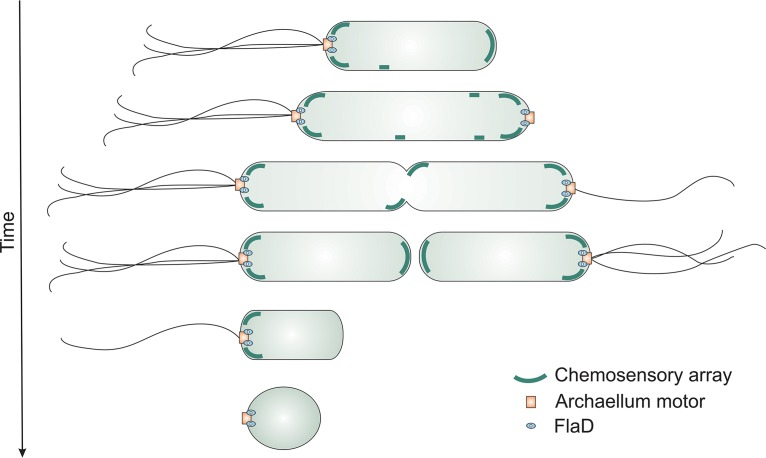
Model of cellular positioning of the motility machinery in the archaeon H. volcanii during different growth phases of the cell. In the early log phase (top), the cells are rod-shaped and possess polar bundles of archaella. Chemosensory arrays (green) are predominantly present at the cell pole, but lateral clusters are also observed, which become polar after cell division. The cells preparing for cell division assemble new archaellum motors at the cell pole to ensure the correct inheritance of the archaella in the daughter cells. After cell division, the cells possess archaella at only a single cell pole. When the cells enter the stationary phase, they lose their archaellum filaments, and the chemosensory arrays are dismantled. FlaD, blue oval; archaellum motor, orange square.

With this work, we provide insights into the spatial and temporal organization of the chemosensory arrays and into the motility structure in archaea. Previously, the spatial positioning of archaella in a few archaea had been reported. For example, electron microscopy (EM) of the euryarchaeal Pyrococcus furiosus showed that the cells possess a thick bundle of archaella (46). However, as the cells are round, it is not clear if this archaellar bundle is anchored at any specific position (46, 47). Multiple archaella of Methanospirillum hungatei were observed by EM to extend from each cell pole of this cylindrically shaped euryarchaeon (48). Cryo-EM revealed that archaella from T.kodakaraensis are present at the cell poles of the rod-shaped cells (49). Finally, over 40 years ago, low-resolution dark-field microscopy was used to study the positions of the archaella in Halobacterium salinarum (3). The rod-shaped cells of this halophilic euryarchaeon were reported to be monopolarly, bipolarly, or lophotrichously archaellated (3). Fluorescence microscopy of fusion proteins has now allowed us to perform the first in-depth study of the intracellular positioning of the archaeal motility machinery using the euryarchaeal model H. volcanii. We found that in the rod-shaped actively dividing cells, the archaella are located exclusively at the cell poles. This might offer a strategy to ensure that both daughter cells inherit an archaellum, as is the case for several polar flagellated bacteria (see review in reference 58). In later growth stages, the cells are barely motile, and the archaellum motor complex is then present at only one location of the rounded cells (Fig. 8). Since the cells are not motile in this stage, FlaD-GFP might be docked on archaellum motor complexes that are “dormant,” i.e., that do not harbor an assembled and actively rotating archaellum filament. Such a situation might reflect the remnants of pilus or flagellar motor complexes found in several bacteria, such as Myxococcus xanthus and Caulobacter crescentus (59–62). For example, M. xanthus produces unipolar type IV pili, which oscillate when the cells reverse direction (59, 63). In this case, several proteins of the pilus motor stay immobile at both poles, while only the ATPases responsible for the extension and retraction of the pili oscillate (59, 63). In addition, in C. crescentus, part of the motor stays present in the membrane, even when no filament is extruded from the cell. In such a case, the cell ejects its flagellum when switching to a nonmotile lifestyle (61, 62). Cryo-EM analysis of whole cells showed that this ejection is an active process, as a plug protein fills the position representing the relic of the flagellar motor, possibly to prevent cell leakage (60). Thus, preservation of a (partial) motor complex of the pili and flagella seems to be a common strategy of the bacteria to ensure rapid subsequent filament assembly and could possibly be used by the archaea as well.

Extracellular signals are transferred to the motility structures from chemosensory receptors organized in arrays in the bacteria and archaea. In H. volcanii, the chemosensory arrays are located at the cell poles, as is the case for the archaellum motor complexes. However, in addition, arrays were also detected along the lateral membranes (see model in Fig. 8). The chemosensory arrays are dynamic, and, after several rounds of cell division, the lateral clusters seem to stay in the polar region (Fig. 8). The presence of large polar chemosensory clusters and multiple smaller lateral clusters resembles the positioning pattern of the chemosensory arrays in E. coli (34, 35, 41). An important difference is that the E. coli lateral clusters are static relative to the local cell wall matrix (34, 35); in contrast, the H. volcanii lateral clusters are highly mobile.

The response regulator CheY and its adaptor protein CheF, which are responsible for transferring signals from the chemosensory arrays to the motility structure, were found primarily at the cell poles in H. volcanii. Sometimes distinct CheY foci at the lateral membranes were observed as well, which, consistent with its signaling role, suggests that CheY is binding to both chemosensory arrays and the base of the archaellum.

In conclusion, these findings show that the positioning of the archaella and the chemosensory arrays is spatially and temporarily regulated. These two macromolecular complexes are assembled only at predefined cellular locations during certain growth phases.

In archaea, the mechanism(s) by which archaella and chemosensory arrays are positioned at specific locations of the cell has not yet been studied in detail. A conical frustum that was observed in cells of the rod-shaped euryarchaeon T.kodakaraensis has been suggested to function as a polar organizing center (49). In bacteria, the mechanisms by which the chemosensory arrays and flagella are positioned in the cell are being mapped in increasingly greater detail. The distribution of the chemosensory arrays in E. coli has been studied in detail, and various explanatory theories, such as stochastic self-assembly (41, 64), membrane curvature sorting (65, 66), and polar preferences of the clusters due to reduced clustering efficiency in the lateral region (35), have been devised. In contrast to E. coli, distinct proteins that are responsible for the placement of the chemosensory arrays at the cell pole have been identified in several bacterial species. In Caulobacter crescentus, the TipN and TipF proteins direct the assembly of the chemosensory arrays to the new pole at a predivisional stage (42). In other species, such as Rhodobacter sphaeroides or *Vibrio* sp., ParA/MinD homologs mediate the interaction with the polar organizing proteins, such as HubP, to position the chemosensory arrays (36–40). In addition to the chemosensory arrays, ParA/MinD homologs are responsible for the correct placement of many macromolecular assemblies in bacteria. They organize cell polarity either by using an oscillation mechanism or by interacting with the polar organizing proteins (36). MinD/ParA homologs are also important to mark the cellular position of the flagella and to control their numbers (36, 58, 67). Several archaea, including H. volcanii, encode one core multiple-MinD/ParA homolog. The organized positioning of the motility machinery observed in H. volcanii reveals the exciting possibility that archaea also use active mechanisms to organize the cellular placement of the macromolecular assemblies.

The model in Fig. 8 summarizes our current knowledge on the spatial and temporal positioning of the archaeal motility machinery. Motile *H. volcanii* cells are rod-shaped and unipolarly archaellated (Fig. 8, row I). Chemosensory arrays have a polar preference, but lateral clusters are present as well. In a predivisional stage, the cell elongates, and a new archaellum motor complex is assembled on the newest pole (Fig. 8, row II). In this stage, the cells can be bipolarly archaellated. Next, chemosensory clusters assemble near the predivision plane prior to cell constriction (Fig. 8, row III). After constriction, the cells are again unipolarly archaellated, while the chemosensory arrays are present at both poles. In the stationary-growth phase, the cells become round and are no longer motile (Fig. 8, row IV). In this stage, (part of) the archaellum motor with FlaD stays present at one cell pole (Fig. 8, row V), while the chemosensory arrays dismantle (Fig. 8, row VI). Since the positioning of the chemosensory arrays and the positioning of the archaella are not interlinked (Fig. 7), their positioning possibly depends on an independent mechanism, such as the MinD/ParA homologs encoded by genes present in the H. volcanii genome.

## MATERIALS AND METHODS

### Growth and genetic manipulation of H. volcanii.

The growth and genetic manipulation of H. volcanii were performed as previously described (12, 68). The primers used for the knockout plasmids based on pTA131 are described in Table S1. Cells were grown in rich YPC medium with Bacto yeast extract, peptone (Oxoid) and Bacto Casamino acids (BD) or in selective CA medium containing only Bacto Casamino acids.

10.1128/mBio.00377-19.8 TABLE S1 Primers used in this study. Download Table S1, DOCX file, 0.01 MB. Copyright © 2019 Li et al. 2019 Li et al. This content is distributed under the terms of the Creative Commons Attribution 4.0 International license.

To express the proteins, plasmids based on pTA1228 (69) were constructed for this study (see Table S2), harboring the pyrE2 cassette. In addition, these plasmids contained mCherry and GFP genes and in-frame restriction sites to enable the expression of N-terminal and C-terminal fluorescent fusion proteins under the control of the tryptophan promoter (see Table S2). Salt-stable GFP and mCherry genes were kindly provided by Duggin et al. (52).

10.1128/mBio.00377-19.9 TABLE S2 Plasmids used in this study. Download Table S2, DOCX file, 0.02 MB. Copyright © 2019 Li et al. 2019 Li et al. This content is distributed under the terms of the Creative Commons Attribution 4.0 International license.

### Strains, plasmids, and primers.

The primer sequences, plasmid sequences, and strains used in this study are listed in Tables S1, S2, and S3, respectively.

10.1128/mBio.00377-19.10 TABLE S3 Strains used in this study. Download Table S3, DOCX file, 0.01 MB. Copyright © 2019 Li et al. 2019 Li et al. This content is distributed under the terms of the Creative Commons Attribution 4.0 International license.

### Motility assays of H. volcanii on semisolid agar plates.

Motility assays were performed as previously described (12). All the plates were inoculated in at least triplicate (containing 3 biological replicates), and the experiment was performed on at least three independent occasions unless stated otherwise.

### Electron microscopy.

CA medium substituted with uracil was inoculated with H. volcanii H26 and HTQ19 cells and grown overnight at 42°C to an optical density (OD) of 0.05. The cells were concentrated using low-speed centrifugation (2,000 × *g*, 10 min) to a theoretical OD of 20 and fixed and prepared for electron microscopy as described previously (12).

### Western blot analysis.

Samples from cultures used for fluorescence microscopy analysis were used to test for the stability and expression of the GFP fusion proteins. Total cell lysates were analyzed with SDS-PAGE (sodium dodecyl sulfate-polyacrylamide gel electrophoresis) and transferred to a polyvinylidene difluoride (PVDF) membrane. After transfer, the membrane was blocked for 2 h at room temperature in 0.2% (wt/vol) I-Block (Thermo Fisher Scientific, MA, USA). Proteins were detected using GFP antibody from rabbit in a mixture with PBST (phosphate-buffered saline with Tween 20; OriGene Technologies Inc., Rockville, MD, USA) (1:1,000) in combination with a secondary anti-rabbit antibody (from goat) coupled to HRP (horseradish peroxidase) (Thermo Fisher Scientific, MA, USA) (1:5,000).

### Fluorescence microscopy.

H.volcanii cultures were grown in CA medium, and, after two serial dilutions performed on subsequent days, the cultures were imaged at an OD of ∼0.03, unless stated otherwise in the text. During the last hour before observation by microscopy, tryptophan was added. The cells were spotted on agar pads composed of 1% agar–18% SW (salt water, containing per liter 144 g NaCl, 21 g MgSO_4_ × 7H_2_O, 18 g MgCl_2_ × 6H_2_O, 4.2 g KCl, and 12 mM Tris HCl [pH 7.3]). The cells were grown and observed on at least three independent occasions, resulting in the analysis of a total of at least several hundred cells.

10.1128/mBio.00377-19.1 TEXT S1 Supplemental methods. Download Text S1, DOCX file, 0.02 MB. Copyright © 2019 Li et al. 2019 Li et al. This content is distributed under the terms of the Creative Commons Attribution 4.0 International license.

For the live imaging of H. volcanii cells to track the mobility of the protein foci, 0.38% agar pads of CA containing 1 mM tryptophan were used in a round 0.17-mm-diameter microscopy dish (Bioptechs), and imaging occurred at 45°C. Images in the PH3 and GFP modes were captured at ×100 magnification every 3 min for 1 h or every 30 min for 16 h.

### Image analysis.

The images were processed using the ImageJ plugin MicrobeJ with the “subcellular localization” function and the “xy cell density” setting (70). Movement of fluorescent foci in the time-lapse image series was characterized by time-space plots generated using the “Surface plotter” function in ImageJ (71).

### Single-cell tracking.

H. volcanii H26 and Δ*cheW* cells were grown as described for the fluorescence microscopy. To follow the *x*-*y* displacement of the cells, phase-contrast images were captured at up to 20 frames/s for 15 s. The swimming trajectories of the cells were determined using Igor pro as previously described (72). Turning angles were measured as the angle between the average of the two angle changes before the reorientation event and the two at the beginning of the new run after the turn.
