# Supplementary material for: Positioning of the Motility Machinery in Halophilic Archaea
Source: mBio. 2019 May 7;10(3):e00377-19. doi: 10.1128/mBio.00377-19 (PMC6509185; doi:10.1128/mBio.00377-19)
Supplement: TABLE S1 [file mBio.00377-19-s0001.docx]

**Supplemental Material and Methods**

**Growth and genetic manipulation of *H. volcanii***

The growth and genetic manipulation of *H. volcanii* were performed as previously described (1). Briefly, depending on the experiment, the cells were grown at 45 °C or 42 °C in either rich YPC medium with Bacto^TM^ yeast extract, peptone (Oxoid, UK) and Bacto^TM^ Casamino acids (BD Biosciences, UK) or in selective CA medium containing only Bacto^TM^ casamino acids. The strains were grown at 45 °C on plates or in liquid cultures shaken at 120 rpm. Gene deletion and gene expression based on selection with uracil in Δ*pyrE2* strains were carried out as described previously (2). The primers used for the knockout plasmids based on pTA131 are described in Table S1. Selection for pop-in occurred on the CA plates, followed by 3 transfers in nonselective YPC medium and the pop-out selection on CA plates complemented with 50 μg/mL 5-FOA and 10 μg/mL uracil. One hundred colonies were streaked on a new YPC plate, grown for two days, and subjected to a colony lift to Zeta-Probe℗ GT Blotting membranes (Biorad). After cell lysis and DNA cross-linking, the DNA was subjected to pre-hybridization and hybridization using a DIG High Prime DNA labeling and detection starter kit II (Roche) according to the manufacturer’s instructions with a DIG-labeled probe of ~100-200 bp annealing in the targeted gene (for primer sequences, see Table S1). Colonies to which the probe did not bind were grown in liquid YPC media, and genomic DNA was isolated as described previously (2). The genomic DNA of several selected mutants was analyzed with PCR using primers that anneal outside of the flanking regions of the deleted gene (see Table S1), and the products formed were compared with those of the wild-type H26 strain on an agarose gel.

To express the proteins, plasmids based on pTA1228 (3) were constructed for this study (see table S2), harboring the pyrE2 cassette. In addition, these plasmids contained mCherry and GFP genes and in-frame restriction sites to enable the expression of N-terminal and C-terminal fluorescent fusion proteins under the control of the tryptophan promoter (see table S2). Salt stable GFP and mCherry genes were kindly provided by Duggin et al (4).

**Strains, plasmids and primers.** The strains, plasmid and primer sequences used in this study can be found in tables S1-S3.

**Motility assays of *H. volcanii* on semisolid agar plates.** Motility assays were performed as previously described (1). Briefly, YPC plates of 0.3% agar containing 1 mM tryptophan were inoculated with 10 µL drops of a preculture of OD ~0.3-0.5. All the strains compared in one experiment were spotted on the same plates. All the plates were inoculated at least in triplicate (containing 3 biological replicates), and the experiment was performed on at least three independent occasions unless stated otherwise. The plates were incubated for 5 days at 45 °C and scanned, and the diameter of the motility halo was subsequently measured. The diameter of the wild-type control on each plate was set to 100% to enable a comparison, and the other diameters were calculated relative to this value and averaged between technical replicates.

**Electron Microscopy.**

A total of 20 mL CA media substituted with uracil was inoculated with *H. volcanii* H26 and HTQ19 cells and grown overnight at 42 ̊C to an OD of 0.05. The cells were concentrated using low speed centrifugation (2000 x g, 10 min) to a theoretical OD of 20 and fixed with 2% (vol/vol) glutaraldehyde and 1% (vol/vol) formaldehyde in CA media at room temperature. The cells were adsorbed on glow-discharged carbon-coated grids with Formvar films. The samples were quickly washed three times with distilled H2O and negatively stained with 2% (wt/vol) uranyl acetate. The cells were imaged using a Philips CM10 transmission EM coupled to a Gatan 792 BioScan camera and the Gatan DigitalMicrograph software.

**Western blot analysis**

The samples were collected from the cultures used for fluorescence microscopy analysis to test for the stability and expression of the GFP fusion proteins. The cell densities of the different cultures were normalized, and all were set to a theoretical OD600=22 in phosphate buffered saline (PBS) buffer. Sodium dodecyl sulfate (SDS) was added to a final concentration of 0.2% (w/v), and the cells were lysed by pipetting up and down until the lysate became clear. Five-fold loading dye (250 mM Tris pH 6.8, 10% SDS(w/v), 10% Dithiothreitol (DTT) (w/v), 50% glycerol (v/v), 50 µg/mL) was added, and the sample was at a final concentration 1X. A total of 5 µL of sample was loaded and analyzed using 11% SDS-PAGE (SDS-polyacrylamide gel electrophoresis). After gel electrophoresis, the proteins were transferred to a PVDF membrane using semidry blotting in blotting buffer (5 mM Tris, 40 mM glycine, 20 (v/v), 0.0375% (v/v)). After the transfer, the membrane was blocked for 2 hours at room temperature in 0.2% (w/v) I-block^TM^ (ThermoFisher Scientific, Massachusetts USA). The membrane was washed 3 x for 15 minutes in PBST (PBS 0.1% Tween) and incubated overnight at 4 ° C in PBST with 1:1000 GFP antibody from rabbits (OriGene Technologies Inc. Rockville, USA). The membrane was incubated again for 15 minutes in 0.2% (w/v) I-block^TM^, followed by 3x 15 minutes washes in PBST buffer. The membrane was incubated for 3 hours in PBST with 1:5000 secondary α-rabbit antibody (from goat) coupled to HRP (horseradish peroxidase) (ThermoFischer Scientific, Massachusetts USA).

**Fluorescence Microscopy**

A total of 5 mL CA of medium was inoculated with *H. volcanii* strains from the plates and grown at a rotating platform overnight at 45 ° C. When the OD was ~0.5 the next day, 5 µl was transferred to an Erlenmeyer flask containing 20 mL CA media that was incubated overnight at 42 ° C until the culture reached OD 0.03 after ~16 hours. During the last hour before observation by microscopy, different concentrations of tryptophan were added to the media to induce protein expression. The cells were spotted on agar pads comprised of 1% agar in 18% SW (containing 144 g NaCl, 21 g MgSO_4_ x 7H_2_O, 18 g MgCl_2_ x 6H_2_O, 4.2 g KCl, and 12 mM Tris HCl (pH 7.3)) and covered with a glass cover slip. The cells were observed with 100x magnification in the phase contrast (PH3) mode and in the GFP/RFP (for mCherry) modes using a Zeiss Axio Observer 2.1 Microscope equipped with a heated XL-5 2000 Incubator running VisiVIEW℗ software. For each experiment, the cells were grown and observed at least on three independent occasions, resulting in the analysis of a total of at least several hundred cells.

For the live imaging of *H. volcanii* cells to track the mobility of the protein foci, 0.38% agar pads made of CA containing 1 mM tryptophan were poured in a round DF 0.17 mm microscopy dish (Bioptechs). The agar pad was removed after drying; the cells were placed under the agar pad, and the lid was placed on the microscopy dish. The microscope chamber was heated at 45 ° C, and the autofocus was enabled. Images in the PH3 and GFP modes were captured at 100 x magnification every 3 minutes for 1 hour. To image the protein foci during the cell division of *H. volcanii,* round agar pads were constructed as described above, and the cells were incubated at 45 ° C for 16 hours. Images were captured every 30 minutes.

**Image analysis**

The images were processed using the ImageJ plugin MicrobeJ (5). The number of foci per cell was counted, and the cells were divided in different bins based on the number of foci they possessed. In addition, the number of cells with the same positioning patterns was calculated as a percentage of the total, resulting in the numbers shown below the fluorescent images in the figures. The parameters of the detection of the fluorescent foci were set to the same levels for all the proteins analyzed. To determine the intracellular localization of the fluorescent foci, the distance between the center of the structure and the cell poles was determined. Since the cells had different lengths and widths, the total cell length and width were set to 100%, and the position of the fluorescent foci was calculated as a percentage of that. The data were plotted using the ‘subcellular localization’ function and the ‘xy cell density setting’ of MicrobeJ as previously described (5). Fluorescent foci movement in the time-lapse image series was characterized by time-space plots generated by the ‘Surface plotter’ function in ImageJ as previously described (6).

**Single-cell tracking.**

*H. volcanii* H26 and Δ*cheW* cells were grown as described for the fluorescence microscopy with the difference that 50 µg/mL of uracil was added to the CA medium. To follow the x-y displacement of the cells, phase-contrast images were captured at up to 20 frames/sec for 15 sec. Swimming trajectories of the cells were determined using Igor pro as previously described (7). Given the trajectory of the cells, ***r* (*t*)** = [*x* (*t*), *y* (*t*)], the swimming velocity ***v* (*t*)** was defined as ***v* (*t*)** = 𝒓 (𝒕 + 𝜟𝒕) − 𝒓 (𝒕) 𝛥𝑡. To eliminate the effect of the Brownian motion of the cells, running averages were calculated over 2 points, which corresponded to 100 msec intervals. Finally, given the two data points, ***r* (*t*)** = [*x* (*t*), *y* (*t*)] and ***r* (*t +* Δ*t*)** = [*x* (*t* + Δ*t*), *y* (*t* + Δ*t*)], we defined the angle against the horizontal axis as *θ* (*t*). If the two successive angle changes *θ*(*t1*)-*θ*(*t0*) and *θ*(*t2*)-*θ*(*t1*) or one successive angle change *θ*(*t1*)-*θ*(*t0*) were over α, that point was identified as the end of the run. A new run begins at three successive angle changes < α. Hence, the minimum duration of a run was set at 300 ms. The threshold α is described as the following equation: α = c Δ*θ*med, where c is the coefficient, and Δ*θ*med is the median directional change. We manually checked the trace and movie to avoid the detection of false events and determined that the optimal value of c is 4. Turning angles were measured as the angle between the average of the two angle changes before the reorientation event and two at the beginning of the new run after the turn.
