# Supplementary material for: Positioning of the Motility Machinery in Halophilic Archaea
Source: mBio. 2019 May 7;10(3):e00377-19. doi: 10.1128/mBio.00377-19 (PMC6509185; doi:10.1128/mBio.00377-19)
Supplement: TABLE S2 [file mBio.00377-19-st002.docx]

**Table S2 – Plasmids used in this study**

| Name | Description |  |
| --- | --- | --- |
| pTA131 | pyrE2 marked deletion plasmid *H.volcanii*. Amp resistance. | (2) |
| pTA1228 | pyrE2 marked protein expression plasmid *H.volcanii*. Amp resistance. Tryptophan inducible. | (3) |
| pIDJL-40 | Expression plasmid *H.volcanii* for C-terminal GFP tagging based on pTA1392. | (4) |
| pSVA3922 | Expression plasmid *H.volcanii* for N-terminal GFP tagging based on pTA1392. | This study |
| pSVA5004 | pTA131 with upstream and downstream flanks of flaD1. Deletion plasmid to delete the flaD1 gene in *H.volcanii*. Deletion cassette is made by ligation via BamHI site of ~500 bp upstream and downstream flanking region. | This study |
| pSVA3919 | pIDJL-40 with *H. volcanii* flaD1. Expression plasmid to express FlaD1-GFP under trp promoter. | This study |
| pSVA5003 | Plasmid for expression of proteins in H.volcanii with a C-terminal GFP-fusion. Created from pTA1228 with primers 7010 and 7011. | This study |
| pSVA3945 | Double expression plasmid to tag proteins with C-terminal GFP and N-terminal mCherry. Created from pSVA5003 with primer 8064 and 8065. | This study |
| pSVA5029 | pTA131 with upstream and downstream flanks of cheW1. Deletion plasmid to delete the cheW1 gene in *H.volcanii*. Deletion cassette is made by ligation via BamHI site of ~500 bp upstream and downstream flanking region. | This study |
| pSVA5031 | pSVA3922 with *H. volcanii* cheW1. Expression plasmid to express CheW1-GFP under trp promoter. | This study |
| pSVA5032 | pIDJL-40 with *H. volcanii* cheW1. Expression plasmid to express GFP-CheW1 under trp promoter. | This study |
| pSVA5078 | pIDJL-40 with *H. volcanii* cheF1. Expression plasmid to express GFP-CheF1 under trp promoter. | This study |
| pSVA5079 | pSVA3922 with *H. volcanii* cheF1. Expression plasmid to express CheF1-GFP under trp promoter. | This study |
| pSVA5603 | pSVA3922 with *H. volcanii* flaD1. Expression plasmid to express GFP-FlaD1 under trp promoter. | This study |
| pSVA5611 | pIDJL-40 with *H. volcanii* cheY. Expression plasmid to express GFP-CheY under trp promoter. | This study |
| pSVA5612 | pSVA3922 with *H.volcanii*_cheY. Expression plasmid to express CheY-GFP under trp promoter. | This study |
| pSVA5616 | pSVA3945 with *H. volcanii*_flaD1. Cloning intermediate to create pSVA5617 | This study |
| pSVA5617 | pSVA5616 with *H. volcanii* _cheW1. Expression plasmid expression plasmid to co-express FlaD1-GFP and mCherry-CheW1 under trp promoter. | This study |
