# Supplementary material for: Positioning of the Motility Machinery in Halophilic Archaea
Source: mBio. 2019 May 7;10(3):e00377-19. doi: 10.1128/mBio.00377-19 (PMC6509185; doi:10.1128/mBio.00377-19)
Supplement: TABLE S3 [file mBio.00377-19-st003.docx]

**Table S3 – Strains used in this study**

| Strains | | | | |  |
| --- | --- | --- | --- | --- | --- |
| Name | **Organism** | **Background strain** | **Genotype** | **Used plasmid** | **Reference** |
| H26 | *H.volcanii* |  | *∆pyrE2* |  | Allers et al. 2004 |
| HTQ19 | *H.volcanii* | H26 | *∆pyrE2 ΔflaD1* | pSVA5004 | This study |
| HTQ32 | *H.volcanii* | H26 | *∆pyrE2 ∆cheY* |  | Quax et al 2018 |
| HTQ63 | *H.volcanii* | H26 | *∆pyrE2 ∆cheW1* | pSVA5029 | This study |
| HTQ403 | *H.volcanii* | H26 | *∆pyrE2 ∆cheF1* |  | Quax et al 2018 |
| HTQ74 | *H.volcanii* | HTQ63 | *∆pyrE2 ∆cheW1:: [GFP-cheW1]* | pSVA5031 | This study |
| HTQ75 | *H.volcanii* | HTQ63 | *∆pyrE2 ∆cheW1:: [cheW1-GFP]* | pSVA5032 | This study |
| HTQ76 | *H.volcanii* | HTQ63 | *∆pyrE2 ∆cheW1:: [pyrE2+]* | pTA1228 | This study |
| HTQ207 | *H.volcanii* | HTQ19 | *∆pyrE2 ΔflaD1::[flaD1-GFP]* | pSVA3919 | This study |
| HTQ319 | *H.volcanii* | HTQ19 | *∆pyrE2 ΔflaD1::[GFP-flaD1]* | pSVA5603 | This study |
| HTQ320 | *H.volcanii* | HTQ19 | *∆pyrE2 ΔflaD1:: [ pyrE2+]* | pTA1228 | This study |
| HTQ355 | *H.volcanii* | HTQ403 | *∆pyrE2 ΔcheF1::[cheF1-GFP]* | pSVA5078 | This study |
| HTQ356 | *H.volcanii* | HTQ403 | *∆pyrE2 ΔcheF1::[GFP-cheF1]* | pSVA5079 | This study |
| HTQ96 | *H.volcanii* | HTQ403 | *∆pyrE2 ∆ cheF1:: [PyrE2+]* | pTA1228 | Quax et al 2018 |
| HTQ364 | *H.volcanii* | HTQ32 | *∆pyrE2 ∆cheY ::[cheY -GFP]* | pSVA5611 | This study |
| HTQ365 | *H.volcanii* | HTQ32 | *∆pyrE2 ∆cheY ::[GFP-cheY]* | pSVA5612 | This study |
| HTQ44 | *H.volcanii* | HTQ32 | *∆pyrE2 ∆cheY ::[ pyrE2+]* | pTA1228 | Quax et al 2018 |
| HTQ379 | *H.volcanii* | HTQ19 | *ΔpyrE2∆flaDΔcheW* | pSVA5029 and pSVA5004 | This study |
| HTQ381 | *H.volcanii* | HTQ379 | *ΔpyrE2∆flaDΔcheW::[flaD1-GFP]* | pSVA3919 | This study |
| HTQ382 | *H.volcanii* | HTQ379 | *ΔpyrE2∆flaDΔcheW::[GFP-CheW1]* | pSVA5031 | This study |
| HTQ383 | *H.volcanii* | HTQ379 | *ΔpyrE2∆flaDΔcheW::[flaD-GFP _mCherry-cheW]* | pSVA5617 | This study |
