# Supplementary material for: Positioning of the Motility Machinery in Halophilic Archaea
Source: mBio. 2019 May 7;10(3):e00377-19. doi: 10.1128/mBio.00377-19 (PMC6509185; doi:10.1128/mBio.00377-19)
Supplement: TEXT S1 [file mBio.00377-19-st001.docx]

**Table S1 – Primers used in this study**

| Name | Sequence | Description |
| --- | --- | --- |
| 7010 | CGGCTAGCATGAGTAAAGGAGAAGAACTTTTCACTGGAGTTGTCCCAATTCTTGTTG | forward primer with NheI site for amplification of GFP for cloning into pTA1228 to create C-terminal GFP fusion plasmid |
| 7011 | GGAATTCTCACTTCTCGAACTGCGGGTGCGACCATTTGTATAGTTCATCCATGCCATG | reverse primer with EcoRI site for amplification of GFP to clone into pTA1228 to create C-terminal GFP fusion plasmid |
| 7167 | GGGGTACCGCCCGGTTTCGTCACGAAG | forward primer with KpnI site for amplification of upstream flank of CheW1 of *H. volcanii* for knock-out construct to clone in pTA131 |
| 7168 | CGGGATCCCTCGGTCGTGATTGCATCTTG | reverse primer with BamHI site for amplification of upstream flank of CheW1 of *H. volcanii* for knock-out plasmid pTA131 |
| 7169 | CGGGATCCGGCGGTTCGACGACCGGCCC | forward primer with BamHI site for amplification of downstream flank of CheW1 of *H. volcanii* for knock-out plasmid pTA131 |
| 7170 | GCTCTAGAGGAGTCGTCCGTCACGACCATCCCCCCGTATGTGGC | reverse primer with XbaI site for amplification of downstream flank of CheW1 of *H. volcanii* for knock-out plasmid pTA131 |
| 7036 | GGAATTCCATATGCGCCGACCGCCGGGTCTC | forward primer with KpnI site for amplification of upstream flank of FlaD1 of *H. volcanii* for knock-out construct to clone in pTA131 |
| 7037 | GGGGTACCGAAGTTCGCGAGGCCGACCCTG | Reverse primer with NdeI site for amplification of upstream flank of FlaD1 of *H. volcanii* for knock-out construct to clone in pTA131 |
| 7038 | GGAATTCCATATGGGGTCACCTCGTTCAGGACG | ​forward primer with NdeI site for amplification of downstream flank of FlaD1 of *H. volcanii* for knock-out construct to clone in pTA131 |
| 7039 | GCTCTAGAGCACGCTGACGACGAGCGGTCTC | Reverse primer with XbaI site for amplification of downstream flank of FlaD1 of *H. volcanii* for knock-out construct to clone in pTA131 |
| 7185 | CCGCCGAAGAAGTACAGGTG | forward primer for amplification of 250 bp probe of CheW from *H.volcanii* |
| 7186 | CCGCCTCGTCTTCGAACTTG | reverse primer for amplification of 250 bp probe of CheW from *H.volcanii* |
| 7195 | TGAGGTCGATCGCCGGTTTG | forward primer to check for CheW KO, annealing outside the flanking regions |
| 7196 | ACGAACCGCGGAATCACGTC | reverse primer to check for CheW KO, annealing outside the flanking regions |
| 7044 | ACGAGCAGGTGTTCGAGTTG | forward primer for amplification of 250 bp probe of FlaD from *H.volcanii* |
| 7045 | TGGACCCGGACGAATACGAC | reverse primer for amplification of 250 bp probe of FlaD from *H.volcanii* |
| 7052 | GCGTGGGTTTCCTTGTACTC | forward primer to check for FlaD KO, annealing outside the flanking regions |
| 7053 | GCAAGGCGCTCAAGCAACTC | reverse primer to check for FlaD KO, annealing outside the flanking regions |
| 7189 | CTAGCTAGCATGAGCGCGGACGAGACCGACGCGGAGG | forward primer for amplification of CheW1 from *H.volcanii* with NheI site for cloning in pSVA3922 |
| 7190 | CGGGATCCTTACTCGATGGCGTGCACCGGCGAAATC | reverse primer for amplification of CheW1 from *H.volcanii* with NheI site for cloning in pSVA3922 |
| 7191 | GGAATTCCATATGAGCGCGGACGAGACCGACGCGGAGG | forward primer for amplification of CheW1 from *H.volcanii* with NdeI site for cloning in pIDLJ40 |
| 7192 | CGGGATCCCTCGATGGCGTGCACCGGCGAAATC | reverse primer for amplification of CheW1 from *H.volcanii* with BamHI site for cloning in pIDLJ40 |
| 7128 | GGAATTCCATATGTACCTGGACCCGGACGAATACGACCCAG | forward primer for amplification of FlaD1 from *H.volcanii* with NdeI site for cloning in pIDJL40 |
| 7129 | CGGGATCCCACCATCGAGGAGAGGCGGG | reverse primer for amplification of FlaD1 from *H.volcanii* with BamHI site for cloning in pIDJL40 |
| 9001 | CTAGCTAGCTACCTGGACCCGGACGAATACGACCCAG | forward primer for amplification of FlaD1 from *H.volcanii* with NheI site for cloning in pSVA3922 |
| 9002 | CGGGATCCTCACACCATCGAGGAGAGGCGGG | reverse primer for amplification of FlaD1 from *H.volcanii* with BamHI site for cloning in pSVA3922 |
| 7231 | GGAATTCCATATGGCAAGCAAGGTACTGGTCGTGGAC | forward primer for amplification of CheY from H.volcanii introducing ndeI site for cloning in pIDJL_40 |
| 7232 | CGGATCCTGCCTCTGCCTGAATCACGTCG | reverse primer for amplification of CheY from H.volcanii introducing BamHI site for cloning in pIDJL_40 |
| 7233 | GGAATTCCATATGAAACCCGGTGAGCAAAAGCTCGGGG | forward primer for amplification of CheF1 from *H.volcanii* with NdeI site for cloning in pIDJL40 |
| 7234 | CGGGATCCCTACTGCTCGTTGATGGCGTCCG | reverse primer for amplification of CheF1 from *H.volcanii* with BamHI site for cloning in pIDJL40 |
| 7235 | CTAGCTAGCATGAAACCCGGTGAGCAAAAGCTCGGGGAC | forward primer for amplification of CheF1 from *H.volcanii* with NheI site for cloning in pSVA3922 |
| 7236 | CGGGATCCCTGCTCGTTGATGGCGTCCG | reverse primer for amplification of CheF1 from *H.volcanii* with BamHI site for cloning in pSVA3922 |
| 8064 | CGGGATCCATGGTGAGCAAGGGCGAGGAGGATAACATGG | Forward primer with BamHI site to amplify mCherry for cloning in pSVA5003 |
| 8065 | ATAAGAATGCGGCCGCTGCATGCATCTTGTACAGCTCGTCCATGC | Reverse primer with NotI site to amplify mCherry for cloning in pSVA5003 |
